# Supplementary material for: A genetic algorithm for self-supervised models of oscillatory neurodynamics
Source: PLoS One. 2026 Aug 5;21(8):e0354021. doi: 10.1371/journal.pone.0354021 (PMC13440876; doi:10.1371/journal.pone.0354021)
Supplement: Supplementary file 2 [file pone.0354021.s004.pdf]

Reviewers' Comments (if peer reviewed):

Reviewer's Responses to Questions
Comments to the Authors:
Please note here if the review is uploaded as an attachment.

We (the authors) provide a point-by-point response to each of the reviewer's remaining concerns below in purple.

Reviewer #1: I thank the authors for addressing and clarifying my previous questions. The addition of the GSDR flowchart improves clarity, and the conceptual structure underlying the algorithm is now clearer.

However, I am now somewhat confused about the central focus of the manuscript. The term "self-supervised" appears pin the title and seems to be positioned as the key contribution of this work. Yet, as I understand it, the self-supervised pathway is only explicitly implemented in the final section involving electrophysiological data (as stated: "Note that to perform the spectral similarity task while allowing only synaptic weights to be trainable is an objective which is more complex than the objectives used in Figs. 1–6. For this task, we used the self-supervision component of GSDR.").
Because the self-supervised component is introduced and evaluated relatively late in the manuscript, it remains unclear whether the primary contribution of the paper is the general GSDR framework or specifically its self-supervised way.

Regarding the focus of the manuscript, we clarify that the primary contribution is the self-supervision framework coupling changes in the supervised and non-supervised path for model training. The reviewer is correct that Figures 1–6 do not have self-supervision. This is foundational and intentional. First, Figures 1-6 establish the computational framework, which includes the Hodgkin-Huxley equations for modeling fundamental single neuron and population dynamics and their training based on metrics (firing rate change, network natural frequency, and spectral power ratios). Second, Figures 1-6 establish the algorithm's core mechanics (convergence, exploration, and parameter sensitivity) in controlled environments. This incremental validation is necessary to prove the framework's robustness prior to applying it to the more complex, high-dimensional spectral similarity task to neurophysiology data shown in the final section (Figures 7&8, Supplemental Figures 4-11 in the revised manuscript), which leverages the self-supervised pathway. We refined the introduction and the caption of Figure 1 to more clearly distinguish between the algorithm's simple form (established in Figures 1-6 based on unsupervised training) and its more complex form (with self-supervised learning in Figures 7&8).

Also, there is a minor issue in Algorithm 1: the pseudocode caption contains incorrect symbols ("??: self-supervision factor, ??: exploration/learning factor"), which should be corrected.

Thanks for noticing this. We have fixed the incorrect symbols issue (i.e, characters that were replaced with "?" when not encoded) in the caption of the algorithm 1.

Regarding the experimental validation, I thank the authors' clarification that a single random seed was used to illustrate the core concept. However, given that the manuscript emphasizes the technical novelty and advantages of the proposed algorithm, evidence of robustness is necessary. Results based on a single realization are insufficient to demonstrate stability and reproducibility. A robust algorithm should not rely on a specific seed.

We did not use a single seed for simulation. We reported the seed used for initial simulation, and subsequent simulations were performed with shuffled seeds. As shown in the Supplemental Figure 8, the explored parameter space varies across trials and sessions. The different data points in Supplemental Figure 8A represent variation across different random seeds and alpha values (with standard deviation across model runs), showing that models with a genetic component and/or with self-supervision (alpha values greater than zero and less than one) outperformed (achieved lower loss values) fully supervised models and the more traditional parameter sweep method. This establishes the robustness of the algorithm and its various components to random initial starting points.

The (depth of the) investigation of the algorithm's properties remains somewhat limited. For example: Is the optimal parameter \alpha, which balances self- and semi-supervision, environment-dependent? what factors determine or regulate this balance? how sensitive are the results to this parameter?

In Supplemental Figure 8, we explored setting alpha to various levels or having the model itself decide the alpha value (the dynamic alpha mode). We assume that differences in alpha values is what the reviewer meant by "environment-dependent". In the dynamic mode, the alpha value can be stochastically changed from trial to trial. We found that the dynamic alpha mode had a similar performance compared to static alpha set to between 0.2-0.8. This shows that low loss values can be achieved with a variety of alpha values (Supplemental Figure 8A). The results are therefore not overly sensitive to the exact alpha value, as models with non-zero alpha (in other words, self-supervised models) consistently outperformed fully supervised models with alpha set to zero.

In the E–I network model, excitatory and inhibitory populations already appear to operate in distinct dynamical regimes prior to training. Whether introducing the self-supervised component alters the learning dynamics differently across these populations?

Thank you for pointing this out, however we did not perform quantitative analysis of population specific (E vs. I) changes during training because we did not have specific hypotheses about how E vs. I synaptic parameter changes affect the oscillatory dynamics. Instead we performed these simulations in an agnostic way to allow the network to self-guide itself through the parameter space to achieve the defined objectives. As an example, in Figure 6 (top-down gamma/beta contextual push-pull) we observed that the model changed synaptic gain differently between the two I populations and the E population (increasing gain on I_slow cells, decreasing gain on I_fast cells, and minimally changing the gain on E cells, parameter values shown in Fig. 6C). The sign of these effects was not pre-specified, yet the model achieved the objective via different learning dynamics across these populations. Additional analysis and simulations would be required to further characterize which population-specific parameter changes allow the model to perform specific tasks (e.g., restricting training on more fine-grained sub-sets of parameters). We plan to analyze such population-specific parameter changes in follow-up work.

Other comments:
Fig. 2B legend still uses “gSDR” (inconsistent terminology). Equations in the "Materials and Methods" section are not well formatted and should be revised. Please ensure consistent font styles for figure labels, particularly within the same figure.

We have refined the figures, symbols and content in the materials and methods section.

Reviewer #3: This paper (Nejat et al.) proposed a novel algorithm, the Genetic Stochastic Delta Rule (GSDR), which is an evolutionary optimization framework designed to bridge high-level self-supervised learning theories with biophysically realistic implementations. Their study demonstrated that the GSDR was able to accomplish a range of simulations, from simple (firing rate matching) to complicated (macaque MT/MST spectrogram matching). The authors provided a promising tool that might be used to study how complex connectivity might emerge in the brain to explain the experimental observations. I like the emphasis on the self-supervision as the consistency with basic neurobiological principles, and I think the authors have done thorough work on building the framework of the model, especially after the revision. However, while the GSDR framework is a technically impressive and timely contribution to the field, the current results raise significant questions regarding the biological realism of the tuned states. I hope these following concerns will be addressed before this article is accepted for publish.

My questions are basically regarding Figures 7-8, about the electrophysiological recording from monkey MT/MST and the following simulations:

1. A notable feature in Figure 7 is that the PSTH/raster plot from these MT/MST neurons had clear "ripples" of firing, and the frequency is very close to the 37.5Hz peak captured in the spectrogram (20 ripples/500ms stimulus duration). In most macaque MT literatures, the gamma oscillation is not a clear, evoked pattern that could be directly observed from the PSTH. It's hard to tell whether the stimulus itself, the filtering techniques applied, or even the experimental hardware (for example, the frequency of the monitor display) from the information the author provided, but I would be a bit suspicious about the spike patterns observed, and it would be helpful if the authors could interpret the data with more thorough considerations.

We understand the reviewer's caution regarding the visibility of these oscillations in the PSTH and that such strong gamma oscillations have not been previously reported. Several factors warrant consideration and indicate that this is a real neuronal signal and is not artifactural. First, we used a large drifting grating stimulus (12 visual degrees radius), which was much larger than typical studies in visual cortex, and also a very high contrast to the stimulus (80%). These parameters likely contributed to a significantly higher excitatory input to areas MT/MST, boosting the strength of the gamma oscillation at a population level. Second, we recorded with high-density probes (128 channel Deep Array, Diagnostic Biochips), capable of recording ~100 neurons simultaneously. The resulting PSTH and raster plots shown in Figure 7 (from 95 neurons) therefore integrate a much larger population of simultaneously recorded single neurons than what was possible with less high-density methods. This may also contribute to the ability of these recordings to more clearly define population rhythms across a larger population of single neurons (which prior methods could only indirectly capture by recording smaller neuronal populations and via the local field potential).

Third, to further show that these gamma oscillations were truly neuronal and not artifactual in nature, we performed additional analyses of the data. We first extended the frequency axis for the reviewer to appreciate that the oscillation is present only in the ~38-40 Hz range (note there is also significant power in the lower frequency theta range), with no peaks in the frequencies that might correspond to screen refresh rate (120 Hz) or electric line noise (60 Hz) - see Supplemental Figure 12. We also characterized the peak gamma frequency of area MT/MST (Supplemental Figure 12, bottom sub-panel) on a trial-by-trial basis, shown as white dots in Supplemental Figure 12, and fit a regression line (teal dotted line). Our observation that the peak gamma frequency increases over the session is inconsistent with a noise source which would be expected to have a non-changing peak frequency. This also replicates earlier work on gamma oscillations in the visual cortex, which showed an increase in the gamma peak frequency from early to later trials in session (Brunet et al., 2014). Finally, in this same session, we also recorded from area PFC with the

same type of recording electrode (upper sub-panel, Supplemental Figure 12). Area PFC did not display the gamma oscillation around ~40Hz, which also rules out a potential artifact due to the screen refresh rate or electronics (PFC recordings are more anterior than the MT/MST recordings and thus closer to the screen). The PFC recordings (calculating the power spectrum based on a population of N=122 simultaneously recorded neurons) had a dominant peak frequency in the beta-band (~20 Hz), which also replicates prior electrophysiology studies showing that beta is a dominant frequency of higher-order cortex (e.g., Lundqvist et al., 2020).

Altogether, these analyses (new Supplemental Figure 12, result section) demonstrate that although our observation of very strong gamma oscillations in the PSTH of neurons in area MT/MST is novel, that it is neurobiological in origin and non-artifactual. These observations strengthen the neurobiological plausibility of our recordings and add to the urgency of understanding the underlying mechanisms via computational modeling, shown in Figure 8 and Supplemental Figures 4-11.

2. It is a related question – the model network seemed to have very high synchrony, no matter in the first few figures or in Figure 8, where the network was trying to capture the beta-to-gamma shift in the recorded MT/MST neurons. Leaving aside the weird 40Hz ripples in the experimental data, will the model tend to “cheat” by simply just fire every neuron together? How well is the network actually capturing the features of the recorded neurons? For example, most MT neuron data reported in the literature could be very noisy, especially when they have different tuning preferences. MT neurons tend to have a baseline firing rate of 5-20Hz (MST might be higher), with a slightly positive pair-wise correlation observed. However, the model showed almost no baseline and a close to 1 correlation. This means one can gain all the information by accessing only one neuron in this network, which is clearly not the case of the brain. It might be due to the neural data this model was trying to mimic, but I tend to believe this to be an artifact of how the model is designed, as this is a consistent signature in the first few figures.

Very high synchrony in computational models of oscillations is indeed a concern for the strong-PING models that we wanted to overcome in our work. Note that our initial models are based on the same underlying model architecture as the E-I networks that simulated gamma oscillations in earlier foundational work of oscillations (Figures 2-3 White et al. 1998, Figure 5 Kopell et al. 2000, Figure 6,13 Börgers et al, 2012)

To quantify the degree of synchrony in the original strong-PING network, we replicated that model in new Supplemental Figure 13 under conditions of weak and strong noise (Supplemental Figure 13). We used kappa as a metric for quantifying synchrony between individual neurons (Rook et al., 1992). As in the classic work and as pointed out by the reviewer, strong PING models were non-physiologically synchronous, with kappa values above 0.5 (Supplemental Figure 13) during periods of strong input drive (between the red

dashed lines). In contrast, compared to strong-PING models, our model had an order of magnitude lower kappa values of 0.058 during visual stimulation (information added to Figure 8). This model is much closer to the kappa values we observed in our empirical recordings of spiking activity in MT/MST (with kappa values of 0.005).

In addition, our model also captured the neurobiological observation that kappa values in the pre-stimulus time period are substantially higher (when beta is dominant) than the stimulus-on period (when gamma is dominant) - see Figure 8 for kappa values of the model and Supplemental Figure 12 for the kappa values of the MT/MST data.

We conclude that our model is a significant step forward in neurobiologically realistic simulations of gamma oscillations compared to classic strong-PING models, but further work is needed to capture the precise synchrony values observed in real data. We also note that our model's additional physiological realism was achieved without explicitly including kappa values in the objective function. Instead, only the relative power spectrum was used as the supervised constraint. Future models would add kappa and other statistics to the model's learning parameters to generate more refined models with higher model-data similarity across additional metrics.

3. The authors stated that "…changes in local synaptic connectivity are sufficient to induce a spectral shift between beta and gamma…". However, changes in local synaptic connectivity would not happen in the scale of 10 milliseconds, in which the spectral shift finished, so I don't think the changes of network parameters reflected changes in the level of connectivity. Therefore, it is more likely to reflect some external input modulations from other networks. I hope the authors will clarify this part of their discussion in the revised article.

We clarify that the rapid (10ms) beta-to-gamma transition reflects the intrinsic dynamics of the pre-tuned biophysical network. No online plasticity or synaptic rewiring occurs during the simulation window; the state resulting from GSDR optimization is inherently capable of these rapid transitions upon stimulus onset. In fact, GSDR acts as a long-scale trial-by-trial tuning algorithm, and rapid plasticity is excluded from models to focus on the algorithm itself and showing its stability, performance and behavior.

Also, some minor catches:
1. In Figure 2, the legend still says "gSDR"
2. Citation 131 didn't seem to be a source of how to locate MT/MST

Thanks for pointing it out. We fixed the unicode-parser which caused several font and character changes (lowercase to upper case, symbols replaced by ? or -). We also fixed the citation shift (132/131).

References cited in letter:

1. Rook AJ, Penning PD. Synchronisation of eating, ruminating and idling activity by grazing sheep. Appl Anim Behav Sci. 1991;32: 157–166. doi:10.1016/S0168-1591(05)80039-5
2. Brunet NM, Bosman CA, Vinck M, Roberts M, Oostenveld R, Desimone R, et al. Stimulus repetition modulates gamma-band synchronization in primate visual cortex. Proc Natl Acad Sci. 2014;111: 3626–3631. doi:10.1073/pnas.1309714111
3. Lundqvist M, Bastos AM, Miller EK. Preservation and Changes in Oscillatory Dynamics across the Cortical Hierarchy. J Cogn Neurosci. 2020;32: 2024–2035. doi:10.1162/jocn_a_01600
4. White JA, Chow CC, Ritt J, No CS-T, Kopell N. Synchronization and Oscillatory Dynamics in Heterogeneous, Mutually Inhibited Neurons.
5. Lee JH, Whittington MA, Kopell NJ. Top-down beta rhythms support selective attention via interlaminar interaction: a model. PLoS Comput Biol. 2013;9: e1003164. doi:10.1371/journal.pcbi.1003164
6. Börgers C, Talei Franzesi G, Lebeau FEN, Boyden ES, Kopell NJ. Minimal size of cell assemblies coordinated by gamma oscillations. PLoS Comput Biol. 2012;8: e1002362. doi:10.1371/journal.pcbi.1002362

Have the authors made all data and (if applicable) computational code underlying the findings in their manuscript fully available?
The PLOS Data policy requires authors to make all data and code underlying the findings described in their manuscript fully available without restriction, with rare exception (please refer to the Data Availability Statement in the manuscript PDF file). The data and code should be provided as part of the manuscript or its supporting information, or deposited to a public repository. For example, in addition to summary statistics, the data points behind means, medians and variance measures should be available. If there are restrictions on publicly sharing data or code —e.g. participant privacy or use of data from a third party—those must be specified.
Reviewer #1: No:
Reviewer #3: Yes

PLOS authors have the option to publish the peer review history of their article (what does this mean?). If published, this will include your full peer review and any attached files.

If you choose “no”, your identity will remain anonymous but your review may still be made public.

Do you want your identity to be public for this peer review? For information about this choice, including consent withdrawal, please see our Privacy Policy.
Reviewer #1: No
Reviewer #3: No

[NOTE: If reviewer comments were submitted as an attachment file, they will be attached to this email and accessible via the submission site. Please log into your account, locate the manuscript record, and check for the action link "View Attachments". If this link does not appear, there are no attachment files.]
