# Supplementary material for: A genetic algorithm for self-supervised models of oscillatory neurodynamics
Source: PLoS One. 2026 Aug 5;21(8):e0354021. doi: 10.1371/journal.pone.0354021 (PMC13440876; doi:10.1371/journal.pone.0354021)
Supplement: Supplementary file 3 [file pone.0354021.s005.pdf]

Overall review

The manuscript touches interesting and important points in current computational neuroscience. Motivated by predictive routing and its mechanisms, the authors focus on modelling and optimization. Specifically, they propose a genetic algorithm (GSDR) to optimize model parameters. This approach is well-justified given the often intractable nature of tuning high-dimensional spiking networks or conducting exhaustive bifurcation analyses.

While the manuscript is promising, the current modeling choices and the scope of the results raise concerns regarding the biological insight provided and the generalizability of the findings. The impact of the work is limited by a lack of comparative analysis across different levels of abstraction. Also, I found the narrative a bit confusing as the authors introduce an algorithm to optimize a model but focus too much on one application.

Given this, I believe it would benefit from a **revision**, and I include specific comments below should the authors decide to prepare a resubmission.

General comments

1. Justification of the Hodgkin-Huxley (HH) Framework

The authors motivate the use of HH models based on their biophysical detail (e.g., voltage-gated ion channels). However, the implementation uses only the standard Na+ and K+ channels, and the optimization is restricted solely to synaptic conductances.

Given that only coupling strength is varied, it is unclear why a 'biophysical' HH model is necessary. I suspect the results would be qualitatively identical in a much simpler excitable system, such as the Izhikevich model.

The authors should justify why the HH detail is required or, more effectively, demonstrate whether these strategies are robust across different levels of abstraction (e.g., Izhikevich or Quadratic Integrate-and-Fire (QIF) networks). I will come back to this point later in the review.

2. Comparative Modeling and Impact

The reliance on a network of 100 HH neurons (approximately 400 differential equations) without comparison to neural mass models (NMM) is a missed opportunity.

Many of the results, specifically the spectral shifts in Figure 7, might be easily captured by canonical NMMs (like Wilson-Cowan) or more recent exact mean-field derivations.

To increase the manuscript's impact and relevance to the whole-brain modeling community, the authors should verify if the mechanisms found in the spiking network translate to an NMM setup, would provide a stronger link between scales.

3. Specificity of the Optimization Results

The authors suggest that the model "discovered" specific synaptic configurations. However, if the optimization is only given one degree of freedom (synaptic conductance), the result is a foregone conclusion rather than an emergent discovery.

The authors should discuss the constraints of the hypothesis. What happens if the model is given more "liberty"? Would it find alternative biological solutions? A more thorough discussion of the hypothesis-driven nature of the GA constraints is necessary.

Specific Comments

Abstract and Summary

The narrative should be reframed. The primary contribution appears to be the application of an optimization algorithm to fit neuronal dynamics, with predictive routing serving as a case study. Currently, the "main point" of the paper feels slightly obscured by the specific application.

Introduction

1. Please specify "(ING)" right after "Interneuron Network Gamma."
2. In the discussion of manual tuning, please clarify which models are being referenced, as not all cited works utilize HH models. Additionally, I suggest avoiding the term "detailed" for point-like neurons.
3. “In this work, we introduce a parameter optimization algorithm to train networks of Hodgkin-Huxley (HH) neurons with biological principles” I am not sure what the authors mean by ‘biological principles’ since the HH used here is the standard one, and so is the methodology applied. If it was a mathematical journal I would understand this naming, but I do not think it is necessary for this audience.

Results

While Fig 2 serves an pedagogical purpose of exemplifying the potential of the stochastic search, Fig 3 and 4 are a feel like trivial example of application, where the authors opt to change the time constant parameter to control the frequency (as expected from the differential equations ruling the system). As this parameter can be deducted from physiology (i.e., there is no reason to change it, as commented later in the text). I suggest moving these to the Appendix and focus on more complex findings.

Spectral push-pull through synaptic self-modulation
This is where the results start to become more interesting. I suggest the authors focus on this section and beyond, deepening the discussion and adding more results.

1. “In one network state, gamma dominates (is more powerful than) beta and in the other network beta dominates gamma” Lack of clarity, define how the frequency was calculated and what it means to ‘dominate’.

2. “The model achieves the conditional objective (Fig. 5, more gamma than beta on condition 1, and more beta than gamma on condition 2). The change in total synaptic

gain (sum of all synaptic connectivity weights) is negligible (Fig. 5A, C), yet the variance of synaptic connection weights has been modulated to match the objective.This shows that synaptic plasticity is capable of modulating the relative spectral response of a neural circuit, without changing time constants or population average/total gain."

How would the system change between conditions? What is the timescale of this change? Is it in the order of the process involved in predictive routing? Could this also be explained by simply targeting different populations with external stimuli?

Narrow-band gamma oscillations are observed in electrophysiology recordings from the primate visual cortex

1. This is the most interesting part of the paper. Fig 7 shows data of gamma oscillations due to stimulus. All the efforts to develop the GSDR are to explain this data. Hence, the paper should be centered about this data and training protocols (like using different models and how GSDR does a better job than other approaches). Moving Figs 3 and 4 to an appendix (which would serve as a detailed tutorial for implementation) would make room for it.

2. The results in Fig 7 can be easily explained with conventional models, neuronal population or neural mass models. The mathematical backbone of these results is that when a parameter is changed, the model passes from a steady-state (no oscillations) to an oscillatory regime. The question we want to answer is, what parameter is changing? The author gives us one hypothesis but it could be discussed deeper.

Self-supervision guides parameter space exploration:

1. Following the last point, what is the parameter that triggers the change in the dynamics of the system? This is related to how the 'presentation of drifting grating stimuli' is modeled and will directly affect the 'conclusion' of the model.
   The authors say:
   "the model was allowed to modulate the synaptic connection conductances"
   What is the motivation behind this modeling choice?
   My problem here is that if you only allow your system one degree of freedom, it is expected that the answer will be related to that degree of freedom. Hence, the starting hypothesis should be better discussed. What other options could be explored?

Discussion

What has GSDR taught… about biophysical models?
This question is not answered here. The authors fail to link the findings with basic concepts of nonlinear dynamical systems such as bifurcations.

What has GSDR taught us about oscillatory dynamics?
References 55 and 96 already found this, so what is the novelty? The authors should leave clear what is the novelty.

What has GSDR taught us about oscillatory shifts?
While these results are interesting, I am afraid they are highly sensitive to the degrees of freedom permitted during optimization. It should be explicitly clarified that the model is essentially conditioned to validate a pre-defined hypothesis. Because the training was constrained within a narrow parameter space, the emergence of these specific results was likely an inevitability of the task design rather than an organic discovery of the algorithm. The discussion would be significantly strengthened by addressing why this specific hypothesis was prioritized and exploring what alternative solutions might emerge if the model were granted greater parametric liberty.

Connections to…
“Thus, we emphasize the importance of making models as similar as possible to neurobiology”

While the authors emphasize the importance of biological realism, the field has frequently demonstrated that complex phenomena - often explored through detailed multicompartmental reconstructions - can be elegantly captured by much simpler models.
The level of abstraction is quite high to claim this biological realism which is not a bad thing given that the authors seem to seek basic mechanisms.

Hence, the manuscript would benefit from acknowledging a 'middle-ground' approach. For instance, theories such as communication-through-coherence have been successfully modeled using Quadratic Integrate-and-Fire (QIF) neurons (e.g., https://doi.org/10.1038/s41467-024-46012-5), proving that biological insight does not strictly require high-dimensional biophysical detail. The authors should frame their work within this broader modeling spectrum rather than asserting an absolute necessity for biophysical grounding.

Given all of this, I think the manuscript would be much more stronger and relevant if the authors would:

1. Reproduce the analysis of the data using a network of Izhikevich or QIF neurons, to see how robust the findings are with respect to model choice. This would increase the reach of the work.
   and/or
2. Reproduce the analysis of the data using a neural mass model (for instance the model of Ref 19 which exhibits slow and fast frequencies, https://doi.org/10.1046/j.1460-9568.2002.01985.x or https://doi.org/10.1103/PhysRevX.5.021028).

Which should be rather feasible. The latter would be especially interesting as it demonstrates the generalizability of the GSDR at a different modelling scale.

Materials and methods

Please write the complete form of Equation 1., i.e., describe the current terms.
