# Supplementary material for: A genetic algorithm for self-supervised models of oscillatory neurodynamics
Source: PLoS One. 2026 Aug 5;21(8):e0354021. doi: 10.1371/journal.pone.0354021 (PMC13440876; doi:10.1371/journal.pone.0354021)
Supplement: Supplementary file 4 [file pone.0354021.s006.pdf]

Response to Reviewers

Dear Editor and Reviewers,

We thank the reviewers for their careful and constructive evaluation of our manuscript. The revised manuscript has been substantially reorganized and reframed in response to the reviews. We now present GSDR as a methodological optimization framework for nonlinear spiking and reduced-spiking neural models, with predictive routing serving as a motivating neuroscience case study rather than the focus.

Major changes include: narrowing the claims around predictive routing; clarifying self-supervision, MCDP, alpha, and genetic selection/deselection; moving simpler pedagogical simulations to the Supplement; centering the Results on spectral push-pull, empirical MT/MST gamma dynamics, spectral-similarity optimization, and model-class robustness; adding reduced-spiking Izhikevich analyses as Supplemental Figs. S16-S18; and revising the Discussion to position Hodgkin-Huxley-style, reduced-spiking, and neural-mass models along a modeling spectrum.

Reviewer #1

This manuscript addresses a critical gap in computational neuroscience: the intensive manual tuning required for biophysically detailed Hodgkin-Huxley neural models. Nejat et al. propose the Genetic Stochastic Delta Rule (GSDR), a hybrid optimization framework that integrates supervised learning with unsupervised representational learning via Mutual Correlation Dependent Plasticity (MCDP). The work aims at demonstrating how this algorithm can bridge the gap between abstract algorithmic theories, such as predictive routing, and their biological implementation by automatically discovering parameters that replicate in-vivo oscillatory rhythms. Notably, the authors validate the model's performance on real electrophysiological data from the macaque visual cortex, showing a robust fit to stimulus-evoked gamma oscillations. Overall, the paper offers a significant technical contribution to the automation of biologically plausible modeling. However, several points regarding the conceptual justification of the "self-supervised" framing, the functional role of the simulated oscillations, and technical inconsistencies in the formalisms require clarification. My specific comments are outlined below.

Major Comments:

1. While the model successfully replicates the frequency shifts (gamma to beta) associated with the Predictive Routing theory, it appears to simulate the spectral phenotype rather than the computational function. The authors note that the model converges to beta rhythms associated with prediction-driven inhibition, but it is not clear if the model is actually performing a functional predictive task or simply being tuned to a specific frequency target. Could the authors clarify if the model performs a functional predictive task (e.g., signaling a mismatch), or if the

link to predictive processing is currently limited to replicating the oscillatory correlates of those states?

Response: We thank the reviewer for identifying this important distinction. We agree that the current manuscript does not demonstrate a complete predictive-processing task. The revised manuscript therefore frames predictive routing as a motivating neuroscience case study, while the primary contribution is methodological: GSDR provides a general optimization framework for fitting nonlinear neural models to electrophysiological objectives. We revised the Introduction and Discussion to state that the present results address spectral and circuit-level phenotypes associated with predictive routing, including beta/gamma state changes, rather than the full computational function of predictive processing. We also added text noting that explicit omission, mismatch, and prediction-error tasks are future applications of the framework.

2. The hybrid nature of GSDR, specifically the inclusion of the unsupervised MCDP path, lacks a direct quantification of why this representational term R is essential for these specific biophysical models. Why is the unsupervised path necessary for reaching the optimal parameter space? Could the authors provide further evidence demonstrating the performance gain of the hybrid model over a purely supervised stochastic delta rule?

Response: We revised the Results and Methods to clarify that GSDR reduces manual parameter tuning by combining objective functions with stochastic exploration, genetic selection/deselection, and an activity-dependent MCDP update term. We now describe MCDP as an activity-dependent representation constrained by the existing model connectivity. In the spectral-similarity task, alpha-controlled mixtures of the supervised term and the MCDP term, particularly intermediate alpha values and the dynamic-alpha implementation, produced better average performance than purely supervised stochastic delta-rule updates and parameter-sweep baselines under matched constraints (Supplementantal Figure 9, 10).

Minor Comments:

3. In the Intrduction, the statement that the brain functions as a self-supervised system "without reliance on external supervision" is somewhat vague. While the brain does not receive "labels" like a machine learning model, it is not an isolated system and relies heavily on external sensory data. It would be helpful to refine this definition to distinguish between "external sensory stimuli" (the data) and "external labels/error signals" (the supervision) to avoid confusion.

Response: We agree and revised the Introduction to separate external sensory data from external labels or experimenter-supplied supervisory signals. The intended point is that biological learning uses sensory input, internal state, and internally generated error or consistency signals without relying primarily on external labels in the supervised-machine-learning sense. We therefore revised the self-supervision framing to emphasize learning from sensory structure and internally generated constraints.

4. The manuscript introduces the Stochastic Delta Rule and Genetic Algorithms as inspirations for GSDR, but a brief conceptual summary of these mechanics in the Results section would improve accessibility for a general neuroscience audience.

Response: We added a short conceptual explanation of both algorithmic ingredients in Algorithm 1. The stochastic delta-rule component proposes random parameter perturbations and uses the resulting change in loss to guide subsequent updates. The genetic component stores the best-performing model state and deselects suboptimal states when the loss exceeds a threshold. We clarified how these components are combined with the MCDP activity-dependent update term through the alpha parameter.

5. The authors claim that GSDR links all three of Marr's levels of analysis. However, the mapping to the "representational" level is the most abstract. Please explicitly state which specific features of the GSDR framework (perhaps the MCDP term) map to the algorithmic/representational level.

Response: We revised the manuscript to reduce the Marr-level framing and to avoid implying that the current model fully instantiates all levels of predictive processing. The revised text treats GSDR as an algorithmic optimization framework that can connect biophysical models to computationally motivated neural objectives, such as spectral power ratios and empirical spectrotemporal targets.

6. The authors refer to the R(t) = MCDP matrix as a "structural representation". Given that MCDP is derived from the similarity of active membrane potentials over time, it is an activity-dependent metric. In a biophysical context, "structural" usually refers to physical connectivity. Would you consider using "functional" or "activity-based" representation to describe this term?

Response: We agree that the phrase "structural representation" could be misleading because, in a biophysical context, structure can imply fixed anatomical connectivity. We revised the terminology to "activity-dependent representation" and, where needed, "activity-dependent representation constrained by the existing model connectivity." We also clarified that MCDP is computed from membrane-potential similarity over time and is not intended to denote anatomical structure.

7. The parameter alpha, which controls the trade-off between supervised and unsupervised paths, is described as dynamic. What specific rule or objective function dictates how alpha is updated? Is it optimized to minimize the loss, or does it follow a predefined random walk?

Response: We clarified the alpha update rule in the Methods (description of Algorithm 1). We compared the performance in the spectral similarity tasks using both static-alpha and dynamic-alpha models (shown in Supplemental Figure 10). In the dynamic-alpha implementation, alpha follows a bounded stochastic random walk rather than a gradient-derived

update or a separate objective function. However, alpha is subject to the same genetic selection/deselection logic as the other trainable quantities: alpha values associated with improved model states can be retained, whereas states exceeding the deselection threshold are reverted to the last selected model state. Thus, alpha is not optimized independently, but its retained trajectory is filtered by model performance.

8. The genetic aspect of the algorithm appears to be the selection and deselection of optimal states. It would be beneficial to explicitly clarify if the deselection step (loss exceeding a limit) is the primary genetic mechanism.

Response: Yes. We revised the Methods to state that the genetic component is the selection/deselection mechanism. GSDR stores the best model state found so far, continues stochastic exploration, and reverts to the stored state when the current loss exceeds a specified deselection threshold. This is the sense in which the algorithm retains fitter parameter states and rejects less fit states.

9. In several examples, such as the E-I firing rate tuning, only one type of parameter (e.g., inhibitory synaptic time constants) is varied. Is GSDR capable of simultaneous multi-parameter optimization across different categories (e.g., tuning conductances and time constants at once)? Clarifying the limits of the parameter search space would be valuable.

Response: We clarified the trainable parameter space for each simulation. GSDR can in principle optimize multiple parameter categories simultaneously, but we did not explore that in this work because it was not part of the hypotheses we considered. We note that in the spectral-similarity task, the trainable space was not one scalar degree of freedom but a high-dimensional matrix of recurrent synaptic conductances, and therefore can be considered as an exploration of a multi-dimensional set of synaptic connectivity parameters.

10. In the Materials and Methods section there are two errors in the internal:
- "parameter alpha" in Eqn. 2, but the equations are labeled 2.1 and 2.2.
- "Stochastic Delta Rule (equation 2.3)," but no equation 2.3 is present in the text

Response: We thank the reviewer for catching these errors. We corrected the equation references so that alpha is consistently described in relation to Equations 2.1 and 2.2, and we removed the reference to the unused equation number.

Reviewer #2
Dear authors,

Please find my review attached.

....
....

Overall review
The manuscript touches interesting and important points in current computational neuroscience. Motivated by predictive routing and its mechanisms, the authors focus on modelling and optimization. Specifically, they propose a genetic algorithm (GSDR) to optimize model parameters. This approach is well-justified given the often intractable nature of tuning high-dimensional spiking networks or conducting exhaustive bifurcation analyses.
While the manuscript is promising, the current modeling choices and the scope of the results raise concerns regarding the biological insight provided and the generalizability of the findings. The impact of the work is limited by a lack of comparative analysis across different levels of abstraction.
Also, I found the narrative a bit confusing. The primary contribution appears to be the application of an optimization algorithm to fit neuronal dynamics, with predictive routing serving as a case study. Currently, the "main point" of the paper feels slightly obscured by the specific application.
I have no preference on what should be focus of the paper, but aspects are quite interesting, but it would help if the authors clarified and maintained a consistent emphasis throughout.
Given this, I believe it would benefit from a revision, and I include specific comments below should the authors decide to prepare a resubmission.

Response: We thank the reviewer for this careful summary and agree that the manuscript needed a clearer central focus. We revised the manuscript to emphasize that the primary contribution is methodological: GSDR is an optimization framework for fitting nonlinear spiking and reduced-spiking neural models to neural objectives. The predictive routing framework is retained as a motivating application because it involves oscillations, spiking, and beta/gamma state changes, but we no longer present the paper as a mechanistic proof of predictive routing. To improve focus, we moved simpler tutorial-style simulations to the Supplement and expanded the empirical spectral-similarity framing.

General comments
1. Justification of the Hodgkin-Huxley (HH) Framework
The authors motivate the use of HH models based on their biophysical detail (e.g., voltage-gated ion channels). However, the implementation uses only the standard Na+ and K+ channels, and the optimization is restricted solely to synaptic conductances.
Given that only coupling strength is varied, it is unclear why a biophysical HH model is necessary. I suspect the results would be qualitatively identical in a much simpler excitable system, such as the Izhikevich model.
The authors should justify why the HH detail is required or, more effectively, demonstrate whether these strategies are robust across different levels of abstraction (e.g., Izhikevich or Quadratic Integrate-and-Fire (QIF) networks). I will come back to this point later in the review.

Response: We agree with the reviewer's central point: for the specific spectral tuning demonstrations in the present manuscript, the full Hodgkin-Huxley level of detail is not strictly

required. We revised the manuscript to state this explicitly and to present HH as one compatible model class, not as a necessary component of GSDR. To address the reviewer directly, we added reduced-spiking Izhikevich simulations as a model-class robustness test. These simulations show that the GSDR framework can be applied beyond the original HH-style model while preserving spike timing, E/I interactions, and stimulus-dependent spectral shifts. We now frame the model choice as dependent on the scientific question: HH-style models are useful for conductance- and ion-channel-level hypotheses, while Izhikevich and other reduced-spiking models provide a compact middle ground (see Discussion).

2. Comparative Modeling and Impact
The reliance on a network of 100 HH neurons (approximately 400 differential equations) without comparison to neural mass models (NMM) is a missed opportunity.
Many of the results, specifically the spectral shifts in Figure 7, might be easily captured by canonical NMMs (like Wilson-Cowan) or more recent exact mean-field derivations.
To increase the manuscript's impact and relevance to the whole-brain modeling community, the authors should verify if the mechanisms found in the spiking network translate to an NMM setup, would provide a stronger link between scales.

Response: We agree that neural mass and mean-field models are important comparison points and that many spectral phenotypes can be reproduced with fewer state variables. We revised the Discussion to acknowledge this. In this revision, we prioritized the Izhikevich reduced-spiking comparison because it directly answers the request for a simpler model class while preserving spike timing, cell-class interactions, recurrent E/I structure, and the ability to define synchrony between neurons. We now state that neural mass models are an important future cross-scale benchmark. Future extensions can embed GSDR-optimized spiking or reduced-spiking models into neural-mass and field/source models, allowing the same optimization logic to be tested across scales.

3. Specificity of the Optimization Results
The authors suggest that the model "discovered" specific synaptic configurations. However, if the optimization is only given one degree of freedom (synaptic conductance), the result is a foregone conclusion rather than an emergent discovery.
The authors should discuss the constraints of the hypothesis. What happens if the model is given more "liberty"? Would it find alternative biological solutions? A more thorough discussion of the hypothesis-driven nature of the GA constraints is necessary.

Response: We agree that the hypothesis-driven constraints of the optimization must be explicit. We revised the manuscript to avoid implying an unconstrained discovery of all possible biological mechanisms. The conductance-only optimization tests a narrower question: whether changes in recurrent synaptic weights can produce the target spectral transition. We chose this specific parameter because prior work in predictive routing had hypothesized this mechanism to engage the beta-gamma push-pull (see Results, page 6). At the same time, the result was not

predetermined by a single scalar degree of freedom; the trainable space contained many individual cell-to-cell synaptic weights. Broader parameter liberty, including intrinsic excitability, synaptic kinetics, neuromodulatory gain, and input targeting is now discussed as future work.

Specific Comments
Introduction
Please specify "(ING)" right after "Interneuron Network Gamma."
In the discussion of manual tuning, please clarify which models are being referenced, as not all cited works utilize HH models. Additionally, I suggest avoiding the term "detailed" for point-like neurons.

Response: We made these changes. "Interneuron Network Gamma" is now followed by "(ING)" at first use.

We removed any implication that manual tuning is only or primarily done for a specific model class. Instead, we now describe the methodological advance afforded by manual tuning, which includes less computational cost and wider exploration of parameter space.

"In this work, we introduce a parameter optimization algorithm to train networks of Hodgkin-Huxley (HH) neurons with biological principles" I am not sure what the authors mean by "biological principles" since the HH used here is the standard one, and so is the methodology applied. If it was a mathematical journal I would understand this naming, but I do not think it is necessary for this audience.

Response: We agree that the phrase "biological principles" was too broad and we replaced it with “biophysically constrained spiking models using evolutionary algorithms”.

Results
While Fig 2 serves an pedagogical purpose of exemplifying the potential of the stochastic search, Fig 3 and 4 are a feel like trivial example of application, where the authors opt to change the time constant parameter to control the frequency (as expected from the differential equations ruling the system). As this parameter can be deducted from physiology (i.e., there is no reason to change it, as commented later in the text). I suggest moving these to the Appendix and focus on more complex findings.

Response: We agree. We moved the simpler pedagogical examples to the Supplement and refocused the main Results on the more central material: the spectral push-pull simulations, the MT/MST empirical gamma response, the spectral-similarity task, and the reduced-spiking Izhikevich model. The moved examples are retained as implementation/tutorial demonstrations.

Spectral push-pull through synaptic self-modulation

This is where the results start to become more interesting. I suggest the authors focus on this section and beyond, deepening the discussion and adding more results.

"In one network state, gamma dominates (is more powerful than) beta and in the other network beta dominates gamma" Lack of clarity, define how the frequency was calculated and what it means to "dominate".

Response: We revised this section for clarity. We removed the term "dominance" and replaced it with the easier to understand phrases that the "gamma-band power exceeded beta-band power" or "the beta/gamma ratios" for clarity.

"The model achieves the conditional objective (Fig. 5, more gamma than beta on condition 1, and more beta than gamma on condition 2). The change in total synaptic gain (sum of all synaptic connectivity weights) is negligible (Fig. 5A, C), yet the variance of synaptic connection weights has been modulated to match the objective. This shows that synaptic plasticity is capable of modulating the relative spectral response of a neural circuit, without changing time constants or population average/total gain."

How would the system change between conditions? What is the timescale of this change? Is it in the order of the process involved in predictive routing? Could this also be explained by simply targeting different populations with external stimuli?

Response: GSDR changes parameters at optimization trials or iterations. Plasticity therefore is assumed to occur on a discrete trial-by-trial timescale, not by rapid within-trial synaptic rewiring. Plasticity changes across this time scale have been proposed by classical models of learning (e.g., The Rescorla-Wagner model) and this time scale of change is consistent with predictive routing proposals (Bastos, Lundqvist et al., 2020, PNAS).

Narrow-band gamma oscillations are observed in electrophysiology recordings from the primate visual cortex

This is the most interesting part of the paper. Fig 7 shows data of gamma oscillations due to stimulus. All the efforts to develop the GSDR are to explain this data. Hence, the paper should be centered about this data and training protocols (like using different models and how GSDR does a better job than other approaches). Moving Figs 3 and 4 to an appendix (which would serve as a detailed tutorial for implementation) would make room for it.

Response: We agree and reorganized the manuscript accordingly.

The results in Fig 7 can be easily explained with conventional models, neuronal population or neural mass models. The mathematical backbone of these results is that when a parameter is changed, the model passes from a steady-state (no oscillations) to an oscillatory regime. The question we want to answer is, what parameter is changing? The author gives us one hypothesis but it could be discussed deeper.

Response: We agree that conventional population or neural mass models can reproduce many spectral transitions. We now acknowledge this explicitly. Our goal is complementary: to test whether a spiking E/I network can reproduce the empirical spectrotemporal response while preserving single-neuron activity, cell-class structure, recurrent synaptic weights, and synchrony measures. We clarified the parameter-level interpretation: during GSDR training, recurrent synaptic conductances are the changing parameters; during the simulated stimulus window, those conductances are fixed and the beta-to-gamma transition is driven by stimulus input interacting with the learned circuit (Page 9).

Self-supervision guides parameter space exploration:
Following the last point, what is the parameter that triggers the change in the dynamics of the system? This is related to how the "presentation of drifting grating stimuli" is modeled and will directly affect the "conclusion" of the model.
The authors say:
"the model was allowed to modulate the synaptic connection conductances"
What is the motivation behind this modeling choice?
My problem here is that if you only allow your system one degree of freedom, it is expected that the answer will be related to that degree of freedom. Hence, the starting hypothesis should be better discussed. What other options could be explored?

Response: Please see our response above. In addition: we manipulated the synaptic conductances as this seemed like the most reasonable approach for how a network could learn to transition between different oscillatory states in stimulus vs. baseline periods. We note that the degrees of freedom in the synaptic connectivity matrix (on the order of 50 x 50) was actually quite high.

Discussion
What has GSDR taught... about biophysical models?
This question is not answered here. The authors fail to link the findings with basic concepts of nonlinear dynamical systems such as bifurcations.

Response: We revised this Discussion section substantially. We describe the result as an optimization and reachability finding: GSDR can identify bounded parameter regimes in which the same spiking or reduced-spiking network expresses different spectral regimes under different input conditions. We added text noting that a formal nonlinear-dynamical-systems analysis would be required to determine whether these transitions correspond to bifurcations, changes in attractor structure, or smoother shifts in oscillatory resonance. That analysis is outside the scope of the current revision.

What has GSDR taught us about oscillatory dynamics?
References 55 and 96 already found this, so what is the novelty? The authors should leave clear what is the novelty.

Response: We agree that the novelty is not the claim that E/I networks can generate beta or gamma rhythms with weak or strong PIN mechanisms; prior work has established that. We revised the Discussion to state the novelty is that GSDR automates the search for parameter regimes that reproduce spectral objectives and empirical spectrotemporal targets, reduces reliance on manual tuning. We therefore present the manuscript as a methodological and benchmarking contribution, not as a new fundamental mechanism of gamma or beta generation. Accordingly, we have removed the section subtitle to reduce the emphasis on novel oscillatory mechanisms.

What has GSDR taught us about oscillatory shifts?
While these results are interesting, I am afraid they are highly sensitive to the degrees of freedom permitted during optimization. It should be explicitly clarified that the model is essentially conditioned to validate a pre-defined hypothesis. Because the training was constrained within a narrow parameter space, the emergence of these specific results was likely an inevitability of the task design rather than an organic discovery of the algorithm. The discussion would be significantly strengthened by addressing why this specific hypothesis was prioritized and exploring what alternative solutions might emerge if the model were granted greater parametric liberty.

Response: We agree and revised the Discussion to be explicit about the hypothesis-driven nature of the optimization. Namely, we investigated whether recurrent synaptic conductance changes, under fixed cellular and synaptic-kinetic assumptions, are sufficient to produce the observed class of spectral shifts. We also added an important qualification: the result was not inevitable merely because conductances were trainable. Under the same broad constraints, fully supervised optimization and parameter-sweep baselines performed worse than hybrid GSDR. Thus, the result supports the usefulness of the GSDR search architecture within a constrained hypothesis space.

Connections to...
"Thus, we emphasize the importance of making models as similar as possible to neurobiology"
While the authors emphasize the importance of biological realism, the field has frequently demonstrated that complex phenomena - often explored through detailed multicompartmental reconstructions - can be elegantly captured by much simpler models.
The level of abstraction is quite high to claim this biological realism which is not a bad thing given that the authors seem to seek basic mechanisms.

Response: We agree and revised this language. We no longer state or imply that the most biophysically detailed model is always the best or necessary choice. Instead, we frame the work along a modeling spectrum: neural mass models provide interpretable population-level dynamics, reduced-spiking models provide efficient cell-level dynamics, and conductance-based models provide access to ion-channel and receptor-level mechanisms. The appropriate model

class depends on the scientific question. For the present paper, the spiking level is useful because it allows us to evaluate spectral matching together with firing rates, sparse participation, and synchrony measures, while the added Izhikevich simulations show that the method is not restricted to HH-style models.

Hence, the manuscript would benefit from acknowledging a "middle-ground" approach. For instance, theories such as communication-through-coherence have been successfully modeled using Quadratic Integrate-and-Fire (QIF) neurons (e.g., https://doi.org/10.1038/s41467-024-46012-5), proving that biological insight does not strictly require high-dimensional biophysical detail. The authors should frame their work within this broader modeling spectrum rather than asserting an absolute necessity for biophysical grounding.

Given all of this, I think the manuscript would be much more stronger and relevant if the authors would:
Reproduce the analysis of the data using a network of Izhikevich or QIF neurons, to see how robust the findings are with respect to model choice. This would increase the reach of the work.

Response: We agree and added a reduced-spiking Izhikevich model as a middle-ground analysis. We chose Izhikevich neurons because the standard parameterization supports compact approximations to regular-spiking excitatory neurons, fast-spiking inhibitory neurons, and low-threshold/SST-like inhibitory neurons, while remaining computationally simpler than HH-style conductance models. The new baseline and stimulus-on Izhikevich results are included as Supplemental Figs. S16-S18. These simulations show that GSDR can be applied beyond the original model class while preserving spike timing, E/I interactions, and stimulus-dependent spectral shifts. We also revised the Discussion to acknowledge reduced models as valuable alternatives rather than treating high-dimensional biophysical detail as a requirement for biological insight.

and/or
Reproduce the analysis of the data using a neural mass model (for instance the model of Ref 19 which exhibits slow and fast frequencies, https://doi.org/10.1046/j.1460-9568.2002.01985.x or https://doi.org/10.1103/PhysRevX.5.021028).

Which should be rather feasible. The latter would be especially interesting as it demonstrates the generalizability of the GSDR at a different modelling scale.

Response: We agree that neural mass models are an important future test of generalizability across scales. In this revision, we focused on the Izhikevich analysis because it directly addresses the request for a simpler model class while retaining spike timing, E/I cell-class structure, and synchrony measures. We agree that expanding GSDR to neural mass models would be interesting and we will evaluate that as future work.

Materials and methods

Please write the complete form of Equation 1., i.e., describe the current terms.

Response: We corrected Equation 1 and expanded the accompanying Methods text to define each current term.
