# Supplementary material for: A genetic algorithm for self-supervised models of oscillatory neurodynamics
Source: PLoS One. 2026 Aug 5;21(8):e0354021. doi: 10.1371/journal.pone.0354021 (PMC13440876; doi:10.1371/journal.pone.0354021)
Supplement: Peer review history

# Author comment: Author response to Decision Letter 0

**DOI:** 10.1371/journal.pone.0354021.r001

**Transfer Alert**

This paper was transferred from another journal. As a result, its full editorial history (including decision letters, peer reviews and author responses) may not be present.

23 Mar 2026

Attachment Submitted filename: rebuttal-plos1-2026-gsdr.pdf

# Peer review: Decision Letter 0

**DOI:** 10.1371/journal.pone.0354021.r002

**Authors:** Jordi Garcia-Ojalvo

26 Apr 2026

PONE-D-26-12323

A genetic algorithm for self-supervised models of oscillatory neurodynamics

PLOS One

Dear Dr. Bastos,

Thank you for submitting your manuscript to PLOS ONE. After careful consideration, we feel that it has merit but does not fully meet PLOS ONE’s publication criteria as it currently stands. Therefore, we invite you to submit a revised version of the manuscript that addresses the points raised during the review process.

Please submit your revised manuscript by Jun 10 2026 11:59PM. If you will need more time than this to complete your revisions, please reply to this message or contact the journal office at plosone@plos.org. When you're ready to submit your revision, log on to https://www.editorialmanager.com/pone/ and select the 'Submissions Needing Revision' folder to locate your manuscript file.

Please include the following items when submitting your revised manuscript:

- A letter that responds to each point raised by the academic editor and reviewer(s). You should upload this letter as a separate file labeled 'Response to Reviewers'.
- A marked-up copy of your manuscript that highlights changes made to the original version. You should upload this as a separate file labeled 'Revised Manuscript with Track Changes'.
- An unmarked version of your revised paper without tracked changes. You should upload this as a separate file labeled 'Manuscript'.

If you would like to make changes to your financial disclosure, please include your updated statement in your cover letter. Guidelines for resubmitting your figure files are available below the reviewer comments at the end of this letter.

If applicable, we recommend that you deposit your laboratory protocols in protocols.io to enhance the reproducibility of your results. Protocols.io assigns your protocol its own identifier (DOI) so that it can be cited independently in the future. For instructions see: https://journals.plos.org/plosone/s/submission-guidelines#loc-laboratory-protocols. Additionally, PLOS ONE offers an option for publishing peer-reviewed Lab Protocol articles, which describe protocols hosted on protocols.io. Read more information on sharing protocols at https://plos.org/protocols?utm_medium=editorial-email&utm_source=authorletters&utm_campaign=protocols.

As the corresponding author, your ORCID iD is verified in the submission system and will appear in the published article. PLOS supports the use of ORCID, and we encourage all coauthors to register for an ORCID iD and use it as well. Please encourage your coauthors to verify their ORCID iD within the submission system before final acceptance, as unverified ORCID iDs will not appear in the published article. *Only*  the individual author can complete the verification step; PLOS staff *cannot*  verify ORCID iDs on behalf of authors.

We look forward to receiving your revised manuscript.

Kind regards,

Jordi Garcia-Ojalvo

Academic Editor

PLOS One

Journal Requirements:

When submitting your revision, we need you to address these additional requirements.

1. Please ensure that your manuscript meets PLOS ONE's style requirements, including those for file naming. The PLOS ONE style templates can be found at

https://journals.plos.org/plosone/s/file?id=wjVg/PLOSOne_formatting_sample_main_body.pdf and

https://journals.plos.org/plosone/s/file?id=ba62/PLOSOne_formatting_sample_title_authors_affiliations.pdf

2. Please note that PLOS One has specific guidelines on code sharing for submissions in which author-generated code underpins the findings in the manuscript. In these cases, we expect all author-generated code to be made available without restrictions upon publication of the work. Please review our guidelines at https://journals.plos.org/plosone/s/materials-and-software-sharing#loc-sharing-code and ensure that your code is shared in a way that follows best practice and facilitates reproducibility and reuse.

3. Thank you for reporting the details of how the primate in your study was cared for. Please also add detail about the dimensions of the cage used, and whether any conspecifics were housed with the primate.

4. Thank you for stating in your Funding Statement:

“This work was supported by NIH-R00MH116100 (AMB), the NSF CAREER grant 2449277 and Vanderbilt startup funds (AMB). The funders had no role in study design, data collection and analysis, decision to publish, or preparation of the manuscript.”

Please provide an amended statement that declares *all* the funding or sources of support (whether external or internal to your organization) received during this study, as detailed online in our guide for authors at http://journals.plos.org/plosone/s/submit-now. Please also include the statement “There was no additional external funding received for this study.” in your updated Funding Statement.

Please include your amended Funding Statement within your cover letter. We will change the online submission form on your behalf.

5. Thank you for stating the following in the Acknowledgments Section of your manuscript:

“This work was supported by NIH-R00MH116100 (AMB), the NSF CAREER grant 2449277 and Vanderbilt startup funds (AMB). We thank Nancy Kopell (and the Cognitive Rhythms and Cognition - CRC team), Homero Esmeraldo, Eli Sennesh for their critical comments to an initial version of this manuscript.”

We note that you have provided funding information that is currently declared in your Funding Statement. However, funding information should not appear in the Acknowledgments section or other areas of your manuscript. We will only publish funding information present in the Funding Statement section of the online submission form.

Please remove any funding-related text from the manuscript and let us know how you would like to update your Funding Statement. Currently, your Funding Statement reads as follows:

“This work was supported by NIH-R00MH116100 (AMB), the NSF CAREER grant 2449277 and Vanderbilt startup funds (AMB). The funders had no role in study design, data collection and analysis, decision to publish, or preparation of the manuscript.”

Please include your amended statements within your cover letter; we will change the online submission form on your behalf.

6. Please include your full ethics statement in the ‘Methods’ section of your manuscript file. In your statement, please include the full name of the IRB or ethics committee who approved or waived your study, as well as whether or not you obtained informed written or verbal consent. If consent was waived for your study, please include this information in your statement as well.

7. If the reviewer comments include a recommendation to cite specific previously published works, please review and evaluate these publications to determine whether they are relevant and should be cited. There is no requirement to cite these works unless the editor has indicated otherwise.

[Note: HTML markup is below. Please do not edit.]

Reviewers' comments:

Reviewer's Responses to Questions

**Comments to the Author**

1. Is the manuscript technically sound, and do the data support the conclusions?

The manuscript must describe a technically sound piece of scientific research with data that supports the conclusions. Experiments must have been conducted rigorously, with appropriate controls, replication, and sample sizes. The conclusions must be drawn appropriately based on the data presented.

Reviewer #1: Yes

Reviewer #2: Yes

**********

2. Has the statistical analysis been performed appropriately and rigorously?

Reviewer #1: Yes

Reviewer #2: Yes

**********

3. Have the authors made all data underlying the findings in their manuscript fully available?

The PLOS Data policy requires authors to make all data underlying the findings described in their manuscript fully available without restriction, with rare exception (please refer to the Data Availability Statement in the manuscript PDF file). The data should be provided as part of the manuscript or its supporting information, or deposited to a public repository. For example, in addition to summary statistics, the data points behind means, medians and variance measures should be available. If there are restrictions on publicly sharing data—e.g. participant privacy or use of data from a third party—those must be specified.

Reviewer #1: Yes

Reviewer #2: Yes

**********

4. Is the manuscript presented in an intelligible fashion and written in standard English?

PLOS ONE does not copyedit accepted manuscripts, so the language in submitted articles must be clear, correct, and unambiguous. Any typographical or grammatical errors should be corrected at revision, so please note any specific errors here.

Reviewer #1: Yes

Reviewer #2: Yes

**********

5. Review Comments to the Author

Please use the space provided to explain your answers to the questions above. You may also include additional comments for the author, including concerns about dual publication, research ethics, or publication ethics. (Please upload your review as an attachment if it exceeds 20,000 characters)

Reviewer #1: This manuscript addresses a critical gap in computational neuroscience: the intensive manual tuning required for biophysically detailed Hodgkin-Huxley neural models. Nejat et al. propose the Genetic Stochastic Delta Rule (GSDR), a hybrid optimization framework that integrates supervised learning with unsupervised representational learning via Mutual Correlation Dependent Plasticity (MCDP). The work aims at demonstrating how this algorithm can bridge the gap between abstract algorithmic theories, such as predictive routing, and their biological implementation by automatically discovering parameters that replicate in-vivo oscillatory rhythms. Notably, the authors validate the model’s performance on real electrophysiological data from the macaque visual cortex, showing a robust fit to stimulus-evoked gamma oscillations. Overall, the paper offers a significant technical contribution to the automation of biologically plausible modeling. However, several points regarding the conceptual justification of the "self-supervised" framing, the functional role of the simulated oscillations, and technical inconsistencies in the formalisms require clarification. My specific comments are outlined below.

Major Comments:

1. While the model successfully replicates the frequency shifts (gamma to beta) associated with the Predictive Routing theory, it appears to simulate the spectral phenotype rather than the computational function. The authors note that the model converges to beta rhythms associated with prediction-driven inhibition, but it is not clear if the model is actually performing a functional predictive task or simply being tuned to a specific frequency target. Could the authors clarify if the model performs a functional predictive task (e.g., signaling a mismatch), or if the link to predictive processing is currently limited to replicating the oscillatory correlates of those states?

2. The hybrid nature of GSDR, specifically the inclusion of the unsupervised MCDP path, lacks a direct quantification of why this representational term R is essential for these specific biophysical models. Why is the unsupervised path necessary for reaching the optimal parameter space? Could the authors provide further evidence demonstrating the performance gain of the hybrid model over a purely supervised stochastic delta rule?

Minor Comments:

3. In the Intrduction, the statement that the brain functions as a self-supervised system "without reliance on external supervision" is somewhat vague. While the brain does not receive "labels" like a machine learning model, it is not an isolated system and relies heavily on external sensory data. It would be helpful to refine this definition to distinguish between "external sensory stimuli" (the data) and "external labels/error signals" (the supervision) to avoid confusion.

4. The manuscript introduces the Stochastic Delta Rule and Genetic Algorithms as inspirations for GSDR, but a brief conceptual summary of these mechanics in the Results section would improve accessibility for a general neuroscience audience.

5. The authors claim that GSDR links all three of Marr’s levels of analysis. However, the mapping to the "representational" level is the most abstract. Please explicitly state which specific features of the GSDR framework (perhaps the MCDP term) map to the algorithmic/representational level.

6. The authors refer to the R(t) = MCDP matrix as a "structural representation". Given that MCDP is derived from the similarity of active membrane potentials over time, it is an activity-dependent metric. In a biophysical context, "structural" usually refers to physical connectivity. Would you consider using "functional" or "activity-based" representation to describe this term?

7. The parameter alpha, which controls the trade-off between supervised and unsupervised paths, is described as dynamic. What specific rule or objective function dictates how alpha is updated? Is it optimized to minimize the loss, or does it follow a predefined random walk?

8. The genetic aspect of the algorithm appears to be the selection and deselection of optimal states. It would be beneficial to explicitly clarify if the deselection step (loss exceeding a limit) is the primary genetic mechanism.

9. In several examples, such as the E-I firing rate tuning, only one type of parameter (e.g., inhibitory synaptic time constants) is varied. Is GSDR capable of simultaneous multi-parameter optimization across different categories (e.g., tuning conductances and time constants at once)? Clarifying the limits of the parameter search space would be valuable.

10. In the Materials and Methods section there are two errors in the internal:

• "parameter alpha" in Eqn. 2, but the equations are labeled 2.1 and 2.2.

• "Stochastic Delta Rule (equation 2.3)," but no equation 2.3 is present in the text

Reviewer #2: Dear authors,

Please find my review attached.

…...............................................

…...............................................

Overall review

The manuscript touches interesting and important points in current computational neuroscience. Motivated by predictive routing and its mechanisms, the authors focus on modelling and optimization. Specifically, they propose a genetic algorithm (GSDR) to optimize model parameters. This approach is well-justified given the often intractable nature of tuning high-dimensional spiking networks or conducting exhaustive bifurcation analyses.

While the manuscript is promising, the current modeling choices and the scope of the results raise concerns regarding the biological insight provided and the generalizability of the findings. The impact of the work is limited by a lack of comparative analysis across different levels of abstraction.

Also, I found the narrative a bit confusing. The primary contribution appears to be the application of an optimization algorithm to fit neuronal dynamics, with predictive routing serving as a case study. Currently, the "main point" of the paper feels slightly obscured by the specific application.

I have no preference on what should be focus of the paper, but aspects are quite interesting, but it would help if the authors clarified and maintained a consistent emphasis throughout.

Given this, I believe it would benefit from a revision, and I include specific comments below should the authors decide to prepare a resubmission.

General comments

1. Justification of the Hodgkin-Huxley (HH) Framework

The authors motivate the use of HH models based on their biophysical detail (e.g., voltage-gated ion channels). However, the implementation uses only the standard Na+ and K+ channels, and the optimization is restricted solely to synaptic conductances.

Given that only coupling strength is varied, it is unclear why a ‘biophysical' HH model is necessary. I suspect the results would be qualitatively identical in a much simpler excitable system, such as the Izhikevich model.

The authors should justify why the HH detail is required or, more effectively, demonstrate whether these strategies are robust across different levels of abstraction (e.g., Izhikevich or Quadratic Integrate-and-Fire (QIF) networks). I will come back to this point later in the review.

2. Comparative Modeling and Impact

The reliance on a network of 100 HH neurons (approximately 400 differential equations) without comparison to neural mass models (NMM) is a missed opportunity.

Many of the results, specifically the spectral shifts in Figure 7, might be easily captured by canonical NMMs (like Wilson-Cowan) or more recent exact mean-field derivations.

To increase the manuscript’s impact and relevance to the whole-brain modeling community, the authors should verify if the mechanisms found in the spiking network translate to an NMM setup, would provide a stronger link between scales.

3. Specificity of the Optimization Results

The authors suggest that the model "discovered" specific synaptic configurations. However, if the optimization is only given one degree of freedom (synaptic conductance), the result is a foregone conclusion rather than an emergent discovery.

The authors should discuss the constraints of the hypothesis. What happens if the model is given more "liberty"? Would it find alternative biological solutions? A more thorough discussion of the hypothesis-driven nature of the GA constraints is necessary.

Specific Comments

Introduction

Please specify "(ING)" right after "Interneuron Network Gamma."

In the discussion of manual tuning, please clarify which models are being referenced, as not all cited works utilize HH models. Additionally, I suggest avoiding the term "detailed" for point-like neurons.

“In this work, we introduce a parameter optimization algorithm to train networks of Hodgkin-Huxley (HH) neurons with biological principles” I am not sure what the authors mean by ‘biological principles’ since the HH used here is the standard one, and so is the methodology applied. If it was a mathematical journal I would understand this naming, but I do not think it is necessary for this audience.

Results

While Fig 2 serves an pedagogical purpose of exemplifying the potential of the stochastic search, Fig 3 and 4 are a feel like trivial example of application, where the authors opt to change the time constant parameter to control the frequency (as expected from the differential equations ruling the system). As this parameter can be deducted from physiology (i.e., there is no reason to change it, as commented later in the text). I suggest moving these to the Appendix and focus on more complex findings.

Spectral push-pull through synaptic self-modulation

This is where the results start to become more interesting. I suggest the authors focus on this section and beyond, deepening the discussion and adding more results.

“In one network state, gamma dominates (is more powerful than) beta and in the other network beta dominates gamma” Lack of clarity, define how the frequency was calculated and what it means to ‘dominate’.

“The model achieves the conditional objective (Fig. 5, more gamma than beta on condition 1, and more beta than gamma on condition 2). The change in total synaptic gain (sum of all synaptic connectivity weights) is negligible (Fig. 5A, C), yet the variance of synaptic connection weights has been modulated to match the objective.This shows that synaptic plasticity is capable of modulating the relative spectral response of a neural circuit, without changing time constants or population average/total gain.”

How would the system change between conditions? What is the timescale of this change? Is it in the order of the process involved in predictive routing? Could this also be explained by simply targeting different populations with external stimuli?

Narrow-band gamma oscillations are observed in electrophysiology recordings from the primate visual cortex

This is the most interesting part of the paper. Fig 7 shows data of gamma oscillations due to stimulus. All the efforts to develop the GSDR are to explain this data. Hence, the paper should be centered about this data and training protocols (like using different models and how GSDR does a better job than other approaches). Moving Figs 3 and 4 to an appendix (which would serve as a detailed tutorial for implementation) would make room for it.

The results in Fig 7 can be easily explained with conventional models, neuronal population or neural mass models. The mathematical backbone of these results is that when a parameter is changed, the model passes from a steady-state (no oscillations) to an oscillatory regime. The question we want to answer is, what parameter is changing? The author gives us one hypothesis but it could be discussed deeper.

Self-supervision guides parameter space exploration:

Following the last point, what is the parameter that triggers the change in the dynamics of the system? This is related to how the ‘presentation of drifting grating stimuli‘ is modeled and will directly affect the ‘conclusion’ of the model.

The authors say:

“the model was allowed to modulate the synaptic connection conductances”

What is the motivation behind this modeling choice?

My problem here is that if you only allow your system one degree of freedom, it is expected that the answer will be related to that degree of freedom. Hence, the starting hypothesis should be better discussed. What other options could be explored?

Discussion

What has GSDR taught… about biophysical models?

This question is not answered here. The authors fail to link the findings with basic concepts of nonlinear dynamical systems such as bifurcations.

What has GSDR taught us about oscillatory dynamics?

References 55 and 96 already found this, so what is the novelty? The authors should leave clear what is the novelty.

What has GSDR taught us about oscillatory shifts?

While these results are interesting, I am afraid they are highly sensitive to the degrees of freedom permitted during optimization. It should be explicitly clarified that the model is essentially conditioned to validate a pre-defined hypothesis. Because the training was constrained within a narrow parameter space, the emergence of these specific results was likely an inevitability of the task design rather than an organic discovery of the algorithm. The discussion would be significantly strengthened by addressing why this specific hypothesis was prioritized and exploring what alternative solutions might emerge if the model were granted greater parametric liberty.

Connections to…

“Thus, we emphasize the importance of making models as similar as possible to neurobiology”

While the authors emphasize the importance of biological realism, the field has frequently demonstrated that complex phenomena - often explored through detailed multicompartmental reconstructions - can be elegantly captured by much simpler models.

The level of abstraction is quite high to claim this biological realism which is not a bad thing given that the authors seem to seek basic mechanisms.

Hence, the manuscript would benefit from acknowledging a 'middle-ground' approach. For instance, theories such as communication-through-coherence have been successfully modeled using Quadratic Integrate-and-Fire (QIF) neurons (e.g., https://doi.org/10.1038/s41467-024-46012-5), proving that biological insight does not strictly require high-dimensional biophysical detail. The authors should frame their work within this broader modeling spectrum rather than asserting an absolute necessity for biophysical grounding.

Given all of this, I think the manuscript would be much more stronger and relevant if the authors would:

Reproduce the analysis of the data using a network of Izhikevich or QIF neurons, to see how robust the findings are with respect to model choice. This would increase the reach of the work.

and/or

Reproduce the analysis of the data using a neural mass model (for instance the model of Ref 19 which exhibits slow and fast frequencies, https://doi.org/10.1046/j.1460-9568.2002.01985.x or https://doi.org/10.1103/PhysRevX.5.021028).

Which should be rather feasible. The latter would be especially interesting as it demonstrates the generalizability of the GSDR at a different modelling scale.

Materials and methods

Please write the complete form of Equation 1., i.e., describe the current terms.

**********

6. PLOS authors have the option to publish the peer review history of their article (what does this mean?). If published, this will include your full peer review and any attached files.

If you choose “no”, your identity will remain anonymous but your review may still be made public.

**Do you want your identity to be public for this peer review?**  For information about this choice, including consent withdrawal, please see our Privacy Policy.

Reviewer #1: No

Reviewer #2: No

**********

[NOTE: If reviewer comments were submitted as an attachment file, they will be attached to this email and accessible via the submission site. Please log into your account, locate the manuscript record, and check for the action link "View Attachments". If this link does not appear, there are no attachment files.]

To ensure your figures meet our technical requirements, please review our figure guidelines: https://journals.plos.org/plosone/s/figures

You may also use PLOS’s free figure tool, NAAS, to help you prepare publication quality figures: https://journals.plos.org/plosone/s/figures#loc-tools-for-figure-preparation.

NAAS will assess whether your figures meet our technical requirements by comparing each figure against our figure specifications.

Attachment Submitted filename: Review_PONE-D-26-12323.pdf

# Author comment: Author response to Decision Letter 1

**DOI:** 10.1371/journal.pone.0354021.r003

15 May 2026

Response to Reviewers

Dear Editor and Reviewers,

We thank the reviewers for their careful and constructive evaluation of our manuscript. The revised manuscript has been substantially reorganized and reframed in response to the reviews. We now present GSDR as a methodological optimization framework for nonlinear spiking and reduced-spiking neural models, with predictive routing serving as a motivating neuroscience case study rather than the focus.

Major changes include: narrowing the claims around predictive routing; clarifying self-supervision, MCDP, alpha, and genetic selection/deselection; moving simpler pedagogical simulations to the Supplement; centering the Results on spectral push-pull, empirical MT/MST gamma dynamics, spectral-similarity optimization, and model-class robustness; adding reduced-spiking Izhikevich analyses as Supplemental Figs. S16-S18; and revising the Discussion to position Hodgkin-Huxley-style, reduced-spiking, and neural-mass models along a modeling spectrum.

Reviewer #1

This manuscript addresses a critical gap in computational neuroscience: the intensive manual tuning required for biophysically detailed Hodgkin-Huxley neural models. Nejat et al. propose the Genetic Stochastic Delta Rule (GSDR), a hybrid optimization framework that integrates supervised learning with unsupervised representational learning via Mutual Correlation Dependent Plasticity (MCDP). The work aims at demonstrating how this algorithm can bridge the gap between abstract algorithmic theories, such as predictive routing, and their biological implementation by automatically discovering parameters that replicate in-vivo oscillatory rhythms. Notably, the authors validate the model's performance on real electrophysiological data from the macaque visual cortex, showing a robust fit to stimulus-evoked gamma oscillations. Overall, the paper offers a significant technical contribution to the automation of biologically plausible modeling. However, several points regarding the conceptual justification of the "self-supervised" framing, the functional role of the simulated oscillations, and technical inconsistencies in the formalisms require clarification. My specific comments are outlined below.

Major Comments:

1. While the model successfully replicates the frequency shifts (gamma to beta) associated with the Predictive Routing theory, it appears to simulate the spectral phenotype rather than the computational function. The authors note that the model converges to beta rhythms associated with prediction-driven inhibition, but it is not clear if the model is actually performing a functional predictive task or simply being tuned to a specific frequency target. Could the authors clarify if the model performs a functional predictive task (e.g., signaling a mismatch), or if the link to predictive processing is currently limited to replicating the oscillatory correlates of those states?

Response: We thank the reviewer for identifying this important distinction. We agree that the current manuscript does not demonstrate a complete predictive-processing task. The revised manuscript therefore frames predictive routing as a motivating neuroscience case study, while the primary contribution is methodological: GSDR provides a general optimization framework for fitting nonlinear neural models to electrophysiological objectives. We revised the Introduction and Discussion to state that the present results address spectral and circuit-level phenotypes associated with predictive routing, including beta/gamma state changes, rather than the full computational function of predictive processing. We also added text noting that explicit omission, mismatch, and prediction-error tasks are future applications of the framework.

2. The hybrid nature of GSDR, specifically the inclusion of the unsupervised MCDP path, lacks a direct quantification of why this representational term R is essential for these specific biophysical models. Why is the unsupervised path necessary for reaching the optimal parameter space? Could the authors provide further evidence demonstrating the performance gain of the hybrid model over a purely supervised stochastic delta rule?

Response: We revised the Results and Methods to clarify that GSDR reduces manual parameter tuning by combining objective functions with stochastic exploration, genetic selection/deselection, and an activity-dependent MCDP update term. We now describe MCDP as an activity-dependent representation constrained by the existing model connectivity. In the spectral-similarity task, alpha-controlled mixtures of the supervised term and the MCDP term, particularly intermediate alpha values and the dynamic-alpha implementation, produced better average performance than purely supervised stochastic delta-rule updates and parameter-sweep baselines under matched constraints (Supplementantal Figure 9, 10).

Minor Comments:

3. In the Intrduction, the statement that the brain functions as a self-supervised system "without reliance on external supervision" is somewhat vague. While the brain does not receive "labels" like a machine learning model, it is not an isolated system and relies heavily on external sensory data. It would be helpful to refine this definition to distinguish between "external sensory stimuli" (the data) and "external labels/error signals" (the supervision) to avoid confusion.

Response: We agree and revised the Introduction to separate external sensory data from external labels or experimenter-supplied supervisory signals. The intended point is that biological learning uses sensory input, internal state, and internally generated error or consistency signals without relying primarily on external labels in the supervised-machine-learning sense. We therefore revised the self-supervision framing to emphasize learning from sensory structure and internally generated constraints.

4. The manuscript introduces the Stochastic Delta Rule and Genetic Algorithms as inspirations for GSDR, but a brief conceptual summary of these mechanics in the Results section would improve accessibility for a general neuroscience audience.

Response: We added a short conceptual explanation of both algorithmic ingredients in Algorithm 1. The stochastic delta-rule component proposes random parameter perturbations and uses the resulting change in loss to guide subsequent updates. The genetic component stores the best-performing model state and deselects suboptimal states when the loss exceeds a threshold. We clarified how these components are combined with the MCDP activity-dependent update term through the alpha parameter.

5. The authors claim that GSDR links all three of Marr's levels of analysis. However, the mapping to the "representational" level is the most abstract. Please explicitly state which specific features of the GSDR framework (perhaps the MCDP term) map to the algorithmic/representational level.

Response: We revised the manuscript to reduce the Marr-level framing and to avoid implying that the current model fully instantiates all levels of predictive processing. The revised text treats GSDR as an algorithmic optimization framework that can connect biophysical models to computationally motivated neural objectives, such as spectral power ratios and empirical spectrotemporal targets.

6. The authors refer to the R(t) = MCDP matrix as a "structural representation". Given that MCDP is derived from the similarity of active membrane potentials over time, it is an activity-dependent metric. In a biophysical context, "structural" usually refers to physical connectivity. Would you consider using "functional" or "activity-based" representation to describe this term?

Response: We agree that the phrase "structural representation" could be misleading because, in a biophysical context, structure can imply fixed anatomical connectivity. We revised the terminology to "activity-dependent representation" and, where needed, "activity-dependent representation constrained by the existing model connectivity." We also clarified that MCDP is computed from membrane-potential similarity over time and is not intended to denote anatomical structure.

7. The parameter alpha, which controls the trade-off between supervised and unsupervised paths, is described as dynamic. What specific rule or objective function dictates how alpha is updated? Is it optimized to minimize the loss, or does it follow a predefined random walk?

Response: We clarified the alpha update rule in the Methods (description of Algorithm 1). We compared the performance in the spectral similarity tasks using both static-alpha and dynamic-alpha models (shown in Supplemental Figure 10). In the dynamic-alpha implementation, alpha follows a bounded stochastic random walk rather than a gradient-derived update or a separate objective function. However, alpha is subject to the same genetic selection/deselection logic as the other trainable quantities: alpha values associated with improved model states can be retained, whereas states exceeding the deselection threshold are reverted to the last selected model state. Thus, alpha is not optimized independently, but its retained trajectory is filtered by model performance.

8. The genetic aspect of the algorithm appears to be the selection and deselection of optimal states. It would be beneficial to explicitly clarify if the deselection step (loss exceeding a limit) is the primary genetic mechanism.

Response: Yes. We revised the Methods to state that the genetic component is the selection/deselection mechanism. GSDR stores the best model state found so far, continues stochastic exploration, and reverts to the stored state when the current loss exceeds a specified deselection threshold. This is the sense in which the algorithm retains fitter parameter states and rejects less fit states.

9. In several examples, such as the E-I firing rate tuning, only one type of parameter (e.g., inhibitory synaptic time constants) is varied. Is GSDR capable of simultaneous multi-parameter optimization across different categories (e.g., tuning conductances and time constants at once)? Clarifying the limits of the parameter search space would be valuable.

Response: We clarified the trainable parameter space for each simulation. GSDR can in principle optimize multiple parameter categories simultaneously, but we did not explore that in this work because it was not part of the hypotheses we considered. We note that in the spectral-similarity task, the trainable space was not one scalar degree of freedom but a high-dimensional matrix of recurrent synaptic conductances, and therefore can be considered as an exploration of a multi-dimensional set of synaptic connectivity parameters.

10. In the Materials and Methods section there are two errors in the internal:

- "parameter alpha" in Eqn. 2, but the equations are labeled 2.1 and 2.2.

- "Stochastic Delta Rule (equation 2.3)," but no equation 2.3 is present in the text

Response: We thank the reviewer for catching these errors. We corrected the equation references so that alpha is consistently described in relation to Equations 2.1 and 2.2, and we removed the reference to the unused equation number.

Reviewer #2

Dear authors,

Please find my review attached.

…...............................................

…...............................................

Overall review

The manuscript touches interesting and important points in current computational neuroscience. Motivated by predictive routing and its mechanisms, the authors focus on modelling and optimization. Specifically, they propose a genetic algorithm (GSDR) to optimize model parameters. This approach is well-justified given the often intractable nature of tuning high-dimensional spiking networks or conducting exhaustive bifurcation analyses.

While the manuscript is promising, the current modeling choices and the scope of the results raise concerns regarding the biological insight provided and the generalizability of the findings. The impact of the work is limited by a lack of comparative analysis across different levels of abstraction.

Also, I found the narrative a bit confusing. The primary contribution appears to be the application of an optimization algorithm to fit neuronal dynamics, with predictive routing serving as a case study. Currently, the "main point" of the paper feels slightly obscured by the specific application.

I have no preference on what should be focus of the paper, but aspects are quite interesting, but it would help if the authors clarified and maintained a consistent emphasis throughout.

Given this, I believe it would benefit from a revision, and I include specific comments below should the authors decide to prepare a resubmission.

Response: We thank the reviewer for this careful summary and agree that the manuscript needed a clearer central focus. We revised the manuscript to emphasize that the primary contribution is methodological: GSDR is an optimization framework for fitting nonlinear spiking and reduced-spiking neural models to neural objectives. The predictive routing framework is retained as a motivating application because it involves oscillations, spiking, and beta/gamma state changes, but we no longer present the paper as a mechanistic proof of predictive routing. To improve focus, we moved simpler tutorial-style simulations to the Supplement and expanded the empirical spectral-similarity framing.

General comments

1. Justification of the Hodgkin-Huxley (HH) Framework

The authors motivate the use of HH models based on their biophysical detail (e.g., voltage-gated ion channels). However, the implementation uses only the standard Na+ and K+ channels, and the optimization is restricted solely to synaptic conductances.

Given that only coupling strength is varied, it is unclear why a biophysical HH model is necessary. I suspect the results would be qualitatively identical in a much simpler excitable system, such as the Izhikevich model.

The authors should justify why the HH detail is required or, more effectively, demonstrate whether these strategies are robust across different levels of abstraction (e.g., Izhikevich or Quadratic Integrate-and-Fire (QIF) networks). I will come back to this point later in the review.

Response: We agree with the reviewer's central point: for the specific spectral tuning demonstrations in the present manuscript, the full Hodgkin-Huxley level of detail is not strictly required. We revised the manuscript to state this explicitly and to present HH as one compatible model class, not as a necessary component of GSDR. To address the reviewer directly, we added reduced-spiking Izhikevich simulations as a model-class robustness test. These simulations show that the GSDR framework can be applied beyond the original HH-style model while preserving spike timing, E/I interactions, and stimulus-dependent spectral shifts. We now frame the model choice as dependent on the scientific question: HH-style models are useful for conductance- and ion-channel-level hypotheses, while Izhikevich and other reduced-spiking models provide a compact middle ground (see Discussion).

2. Comparative Modeling and Impact

The reliance on a network of 100 HH neurons (approximately 400 differential equations) without comparison to neural mass models (NMM) is a missed opportunity.

Many of the results, specifically the spectral shifts in Figure 7, might be easily captured by canonical NMMs (like Wilson-Cowan) or more recent exact mean-field derivations.

To increase the manuscript's impact and relevance to the whole-brain modeling community, the authors should verify if the mechanisms found in the spiking network translate to an NMM setup, would provide a stronger link between scales.

Response: We agree that neural mass and mean-field models are important comparison points and that many spectral phenotypes can be reproduced with fewer state variables. We revised the Discussion to acknowledge this. In this revision, we prioritized the Izhikevich reduced-spiking comparison because it directly answers the request for a simpler model class while preserving spike timing, cell-class interactions, recurrent E/I structure, and the ability to define synchrony between neurons. We now state that neural mass models are an important future cross-scale benchmark. Future e

Attachment Submitted filename: PLOS_ONE_full_review_response_rebuttal_corrected.docx (1).pdf

# Peer review: Decision Letter 1

**DOI:** 10.1371/journal.pone.0354021.r004

**Authors:** Jordi Garcia-Ojalvo

2 Jul 2026

A genetic algorithm for self-supervised models of oscillatory neurodynamics

PONE-D-26-12323R1

Dear Dr. Bastos,

We’re pleased to inform you that your manuscript has been judged scientifically suitable for publication and will be formally accepted for publication once it meets all outstanding technical requirements.

Within one week, you’ll receive an e-mail detailing the required amendments. When these have been addressed, you’ll receive a formal acceptance letter and your manuscript will be scheduled for publication.

An invoice will be generated when your article is formally accepted. Please note, if your institution has a publishing partnership with PLOS and your article meets the relevant criteria, all or part of your publication costs will be covered. Please make sure your user information is up-to-date by logging into Editorial Manager at Editorial Manager® and clicking the ‘Update My Information' link at the top of the page. For questions related to billing, please contact billing support.

If your institution or institutions have a press office, please notify them about your upcoming paper to help maximize its impact. If they’ll be preparing press materials, please inform our press team as soon as possible -- no later than 48 hours after receiving the formal acceptance. Your manuscript will remain under strict press embargo until 2 pm Eastern Time on the date of publication. For more information, please contact onepress@plos.org.

Kind regards,

Jordi Garcia-Ojalvo

Academic Editor

PLOS One

Additional Editor Comments (optional):

Reviewers' comments:

Reviewer's Responses to Questions

**Comments to the Author**

1. If the authors have adequately addressed your comments raised in a previous round of review and you feel that this manuscript is now acceptable for publication, you may indicate that here to bypass the “Comments to the Author” section, enter your conflict of interest statement in the “Confidential to Editor” section, and submit your "Accept" recommendation.

Reviewer #1: All comments have been addressed

Reviewer #2: All comments have been addressed

**********

2. Is the manuscript technically sound, and do the data support the conclusions?

The manuscript must describe a technically sound piece of scientific research with data that supports the conclusions. Experiments must have been conducted rigorously, with appropriate controls, replication, and sample sizes. The conclusions must be drawn appropriately based on the data presented.

Reviewer #1: Yes

Reviewer #2: Yes

**********

3. Has the statistical analysis been performed appropriately and rigorously?

Reviewer #1: Yes

Reviewer #2: N/A

**********

4. Have the authors made all data underlying the findings in their manuscript fully available?

The PLOS Data policy requires authors to make all data underlying the findings described in their manuscript fully available without restriction, with rare exception (please refer to the Data Availability Statement in the manuscript PDF file). The data should be provided as part of the manuscript or its supporting information, or deposited to a public repository. For example, in addition to summary statistics, the data points behind means, medians and variance measures should be available. If there are restrictions on publicly sharing data—e.g. participant privacy or use of data from a third party—those must be specified.

Reviewer #1: Yes

Reviewer #2: Yes

**********

5. Is the manuscript presented in an intelligible fashion and written in standard English?

PLOS ONE does not copyedit accepted manuscripts, so the language in submitted articles must be clear, correct, and unambiguous. Any typographical or grammatical errors should be corrected at revision, so please note any specific errors here.

Reviewer #1: Yes

Reviewer #2: Yes

**********

6. Review Comments to the Author

Please use the space provided to explain your answers to the questions above. You may also include additional comments for the author, including concerns about dual publication, research ethics, or publication ethics. (Please upload your review as an attachment if it exceeds 20,000 characters)

Reviewer #1: Major comment 1: The authors agreed that the model does not perform a complete predictive task and they reframed the Predictive Routing as a "motivating neuroscience case study" rather than a functional proof.

Major comment 2: The authors clarified MCDP as an "activity-dependent representation". They also added Supplemental Figures 9 and 10 to demonstrate that dynamic/intermediate alpha values outperform purely supervised rules.

Minor comment 3: The terminology is now much more precise for a neuroscience context.

Minor comment 4: The authors added a conceptual explanation to Algorithm 1, detailing how random perturbations combine with a threshold-based state retention system.

Minor comment 5: They removed the Marr-level framing entirely.

Minor comment 6: The authors replaced "structural representation" with "activity-dependent representation" throughout the text.

Minor comment 7: They clarified that alpha follows a bounded stochastic random walk. It is not optimized independently but is filtered by the same genetic selection/deselection rules as other variables.

Minor comment 8: Resolved

Minor comment 9: The authors clarified that GSDR can optimize multiple parameter categories. They also noted that their spectral-similarity task serves as a high-dimensional example.

Minor comment 10: Resolved

Reviewer #2: Dear authors,

Thank you for addressing all my comments.

In my view the manuscript is now suitable for publication.

Best regards.

**********

7. PLOS authors have the option to publish the peer review history of their article (what does this mean?). If published, this will include your full peer review and any attached files.

If you choose “no”, your identity will remain anonymous but your review may still be made public.

**Do you want your identity to be public for this peer review?**  For information about this choice, including consent withdrawal, please see our Privacy Policy.

Reviewer #1: No

Reviewer #2: No

**********

# Editor report: Acceptance letter

**DOI:** 10.1371/journal.pone.0354021.r005

**Authors:** Jordi Garcia-Ojalvo

PONE-D-26-12323R1

PLOS One

Dear Dr. Bastos,

I'm pleased to inform you that your manuscript has been deemed suitable for publication in PLOS One. Congratulations! Your manuscript is now being handed over to our production team.

At this stage, our production department will prepare your paper for publication. This includes ensuring the following:

* All references, tables, and figures are properly cited

* All relevant supporting information is included in the manuscript submission,

* There are no issues that prevent the paper from being properly typeset

You will receive further instructions from the production team, including instructions on how to review your proof when it is ready. Please keep in mind that we are working through a large volume of accepted articles, so please give us a few days to review your paper and let you know the next and final steps.

Lastly, if your institution or institutions have a press office, please let them know about your upcoming paper now to help maximize its impact. If they'll be preparing press materials, please inform our press team within the next 48 hours. Your manuscript will remain under strict press embargo until 2 pm Eastern Time on the date of publication. For more information, please contact onepress@plos.org.

You will receive an invoice from PLOS for your publication fee after your manuscript has reached the completed accept phase. If you receive an email requesting payment before acceptance or for any other service, this may be a phishing scheme. Learn how to identify phishing emails and protect your accounts at https://explore.plos.org/phishing.

If we can help with anything else, please email us at customercare@plos.org.

Thank you for submitting your work to PLOS ONE and supporting open access.

Kind regards,

PLOS ONE Editorial Office Staff

on behalf of

Dr. Jordi Garcia-Ojalvo

Academic Editor

PLOS One
